# Machine learning to help researchers evaluate biases in clinical trials: a prospective, randomized user study

**DOI:** 10.1186/s12911-019-0814-z

**Published:** 2019-05-08

**Authors:** Frank Soboczenski, Thomas A. Trikalinos, Joël Kuiper, Randolph G. Bias, Byron C. Wallace, Iain J. Marshall

**Affiliations:** 1 School of Population Health & Environmental Sciences, Faculty of Life Sciences and Medicine, King’s College London, 3rd Floor, Addison House, Guy’s Campus, London, SE1 1UL UK; 2 Center for Evidence Synthesis in Health, Brown University, Providence, USA; 3 Vortext Systems, Groningen, Netherlands; 4 School of Information, University of Texas at Austin, Austin, USA; 5 Khoury College of Computer Sciences, Northeastern University, Boston, USA

## Abstract

**Objective:**

Assessing risks of bias in randomized controlled trials (RCTs) is an important but laborious task when conducting systematic reviews. RobotReviewer (RR), an open-source machine learning (ML) system, semi-automates bias assessments. We conducted a user study of RobotReviewer, evaluating time saved and usability of the tool.

**Materials and methods:**

Systematic reviewers applied the Cochrane Risk of Bias tool to four randomly selected RCT articles. Reviewers judged: whether an RCT was at low, or high/unclear risk of bias for each bias domain in the Cochrane tool (Version 1); and highlighted article text justifying their decision. For a random two of the four articles, the process was semi-automated: users were provided with ML-suggested bias judgments and text highlights. Participants could amend the suggestions if necessary. We measured time taken for the task, ML suggestions, usability via the System Usability Scale (SUS) and collected qualitative feedback.

**Results:**

For 41 volunteers, semi-automation was quicker than manual assessment (mean 755 vs. 824 s; relative time 0.75, 95% CI 0.62–0.92). Reviewers accepted 301/328 (91%) of the ML Risk of Bias (RoB) judgments, and 202/328 (62%) of text highlights without change. Overall, ML suggested text highlights had a recall of 0.90 (SD 0.14) and precision of 0.87 (SD 0.21) with respect to the users’ final versions. Reviewers assigned the system a mean 77.7 SUS score, corresponding to a rating between “good” and “excellent”.

**Conclusions:**

Semi-automation (where humans validate machine learning suggestions) can improve the efficiency of evidence synthesis. Our system was rated highly usable, and expedited bias assessment of RCTs.

## Background and significance

Evidence-Based Medicine (EBM) aspires to inform patient care using the entirety of the available evidence [1]. Unfortunately, evidence — in particular, findings from clinical trials — is generally disseminated in unstructured journal articles. This vast and rapidly expanding unstructured evidence base imposes substantial burden on researchers aiming to synthesize all findings relevant to a given clinical question [2].

A key step in appraising the evidence is assessing potential risks of biases introduced by problems in trial design, analysis, or execution [3]. Such biases may lead to over- or underestimation of intervention effects. The Cochrane Risk of Bias (RoB) tool [3] was designed to standardize and aid risk of bias assessment for randomized controlled trials (RCTs). It structures assessments in six domains, namely “selection” (the RoB tool’s term for confounding), performance, detection, attrition, selective reporting, and other biases. It encourages reviewers to consider specific attributes of study design, conduct, analysis, and reporting to inform judgments concerning whether the risk of bias in each domain is *low*, *high*, or *unclear*. These attributes were chosen on the basis of theory and empirical data, and include the adequacy of randomization sequence generation and allocation concealment (for selection bias), blinding of participants and study personnel (for performance bias), blinding of outcome assessors (for detection bias), incomplete outcome data (for attrition bias), as well as other items suggestive of selective reporting and other biases. Because bias judgments are subjective, reviewers are asked to record supporting *rationales* — typically these are verbatim snippets from the article that support the assessment.

In practice, risk of bias assessments are performed together with data extraction. It has been estimated that experienced systematic reviewers devote around 20 min per article, on average [4]. Systematic reviews typically include several dozen to a few hundreds of papers, and most tasks (including bias assessment) are performed independently by two reviewers. Therefore, even modest reductions to the time required to perform risk of bias assessments would be impactful.

Machine learning (ML) and natural language processing (NLP) technologies have the potential to expedite biomedical evidence synthesis via semi-automation of time-intensive tasks [5]. A small body of work on developing such methods has emerged [6, 7]. However, almost all of this work has considered retrospective evaluation of ML/NLP methods applied to existing datasets, providing only indirect evidence of the utility of such methods for EBM. The extent to which ML technologies might benefit systematic reviewers in practice has yet to be rigorously assessed. We address this gap by presenting a pragmatic randomized user study to evaluate the practical utility of semi-automation for expediting biomedical evidence syntheses.

## Materials and methods

### The RobotReviewer system

RobotReviewer is an open-source^1^ ML system developed by our team, designed to automatically extract key data items from reports of clinical trials. We have described this tool in detail elsewhere [8–11]. Here we use it for its ability to automate the assessment of risks of bias using the Cochrane tool [3]. We focus on four common aspects: random sequence generation (RSG), allocation concealment (AC), blinding of participants and personnel (BPP), and blinding of outcome assessors (BOA). These domains can be assessed solely based on the information included in the paper and do not require numerical calculations (as needed, e.g., to assess attrition bias). The original tool (Version 1) provides three possible ratings: “low”, “high”, or “unclear” risk of bias. We dichtomise bias judgements into the two classes “low”, and “high/unclear” to simplify the ML model, and additionally because this is often how bias assessments are used in practice (e.g. systematic reviews often conduct sensitivity analyses of the ‘low’ risk of bias articles only) [8].

RobotReviewer provides bias judgments and snippets of text from the article supporting these (*rationales*). Our focus is on a *semi*-automated workflow in which RobotReviewer makes suggestions to human reviewers, who then have the opportunity to accept or amend these. This approach has the potential to expedite synthesis without sacrificing reliability.

### Participants

We aimed to recruit volunteer researchers with experience in conducting systematic reviews and assessing clinical trial reports for biases. Participants were invited by email and Twitter advertisements via systematic review researcher networks (including Cochrane Crowd,^2^ the EPPI Centre,^3^ the Society for Research Synthesis Methods^4^). Interested participants emailed the authors, and those deemed to have sufficient experience were sent the study URL. We collected the following information for each participant: (i) How many systematic review tasks they had performed; (ii) How many systematic reviews they had completed and; (iii) Whether they had experience using the Cochrane RoB tool. No other individual data was collected.

### Models

We used the ML approach we have described in detail elsewhere [9], and the same version that is freely available on our website^5^ and as open-source software. Briefly, we use an ensemble comprising (1) A multi-task linear-kernel Support Vector Machine (SVM) variant [10]; and, (2) A *Convolutional Neural Network* (RA-CNN) [12]. Both models are *rationale-augmented*, that is, they simultaneously classify articles (as being at *high/unclear* or *low* risk of bias) and identify text snippets in the article that most strongly support this classification.

**Fig. 1:**
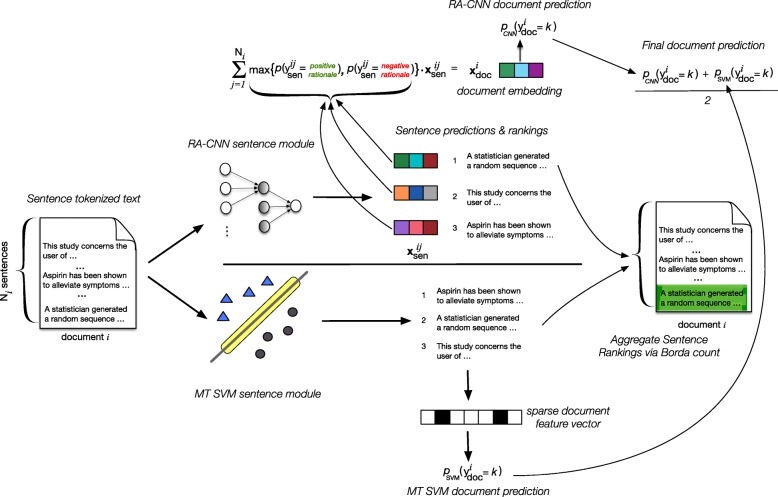
A schematic of our approach to automated risk of bias prediction and supportive rationale extraction. This is an ensemble composed of two models that we have described at length elsewhere: a linear kernel multi-task SVM variant (MT-SVM; bottom) [10], and a rationale-augmented CNN (RA-CNN; top-*k*) [11]

A schematic of the ensemble model is shown in Fig. 1. The constituent models were trained using corpora derived from the Cochrane Database of Systematic Reviews (CDSR). These were constructed by matching individual study records in the CDSR to corresponding full-text journal articles. The CDSR records typically include document judgments for the domains of interest, and often record rationales supporting each bias assessment. Frequently these are verbatim quotes from the journal article. We use heuristics to match these to text snippets in the full-text article. This is a noisy process because quotes are not always perfectly transcribed by review authors, and automatic text extraction from PDFs does not always yield clean output. This may thus be viewed as an instance of *distant supervision* [13–15].

The final training set size varied across domains, numbering ~ 3000 to ~ 12,000 labeled full-text articles per domain. We describe the training corpora and procedures used to train the linear and neural RoB models elsewhere [8, 11].

**Fig. 2:**
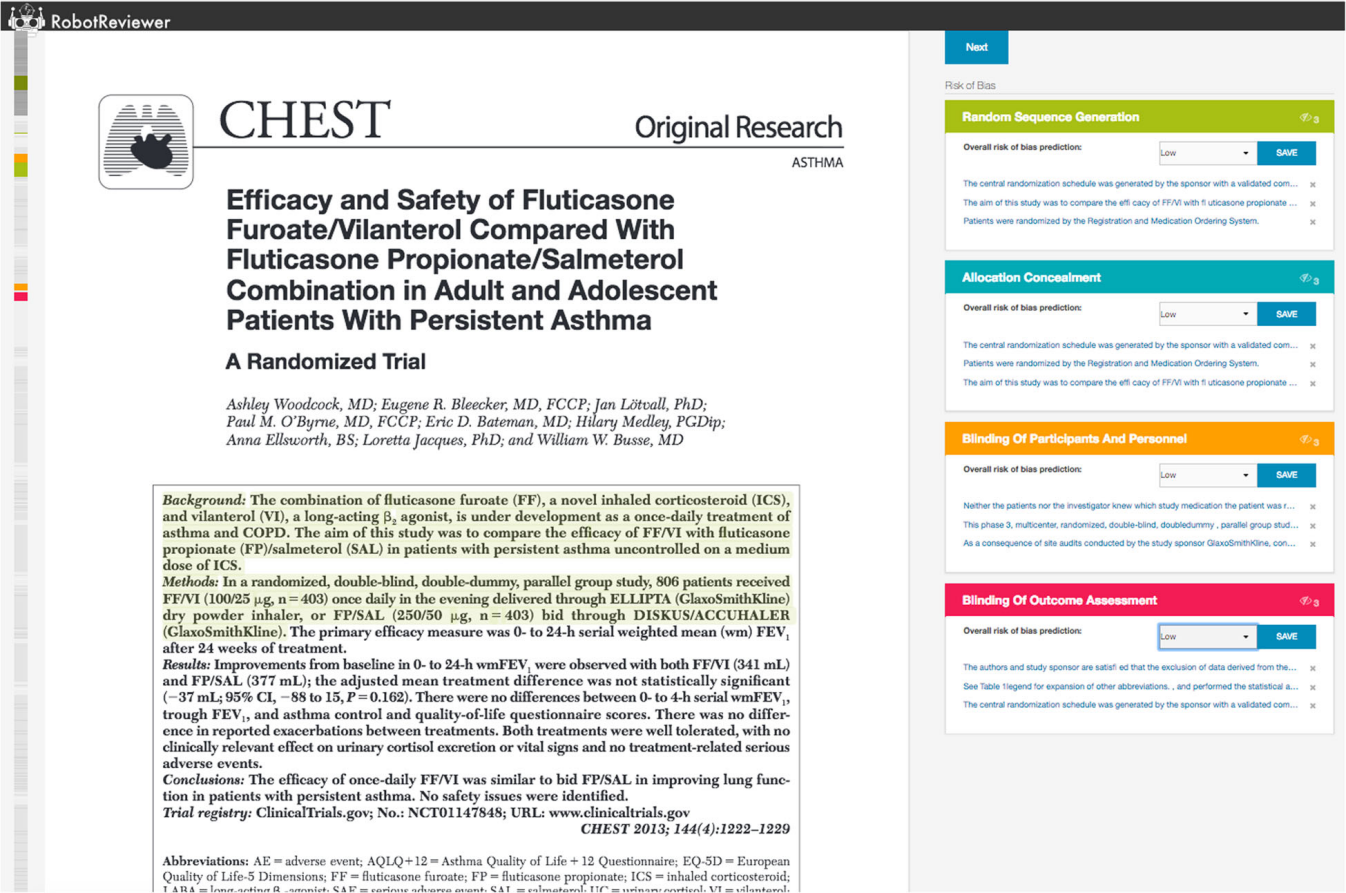
The RobotReviewer web interface. Screenshot of the RobotReviewer web interface, as used by study participants, with this example showing machine learning suggestions. Participants viewed the article (left), and completed the risk of bias assessment (right)

To allow users to interact with, edit, and correct RobotReviewer’s suggestions, we developed a web interface (Fig. 2) [16]. This comprises a PDF viewer (left), and a summary of the automatic RoB assessments (right). Rationales are highlighted directly in the PDF, and the user can navigate between them by clicking on the bias judgments on the right panel.

### Evaluation design

This study was run entirely online. Participants read a study information page and were asked to confirm consent to participate before starting. After consent, an instructional video was presented to participants.^6^

To ensure the task somewhat reflected the breadth of real-world practice, we selected 52 articles randomly sampled from studies that were both (a) included in systematic reviews published in the Cochrane Library, and (b) were available with an open access license. Of these 52 articles, each participant was assigned four for which they were tasked with conducting risk of bias assessment. For each article, participants were asked to assign a risk of bias (either *low*, or *high/unclear*) for the four bias domains enumerated above (RSG, AC, BPP, BOA), and to mark text in the PDF that supported these judgments. We limited each participant to four articles because pilot experiments indicated that the task was time consuming, and that a greater number per participant would impede trial participation as well as increase the risk of fatigue effects coming into play.

A within-subject design was used in which each participant was shown two trial reports with ML suggestions (the semi-automated task), and two trial reports without (the manual task). The order in which these studies were presented was randomized. With the semi-automated task, bias judgments and rationales were automatically populated; participants had only to optionally correct and approve them. In the manual condition, participants completed the task without machine assistance, starting from an unannotated PDF shown in the same interface. No time limit to complete the task was imposed.

**Fig. 3:**
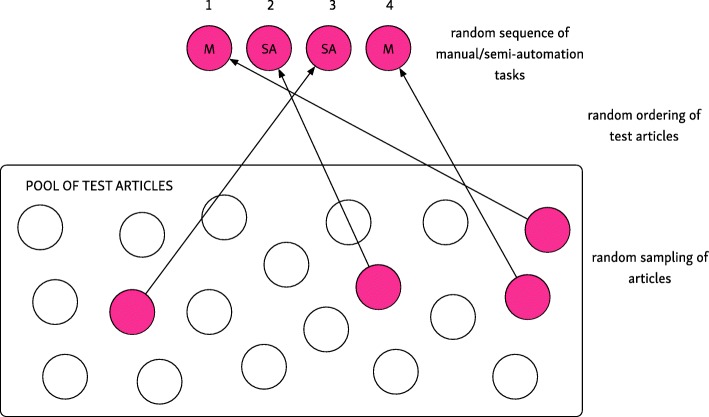
Randomization strategy. For each participant, we randomized: the selection and order of test articles, and the sequence which the two semi-automation and two manual tasks were presented

To minimize effects of individual article difficulty, user fatigue, and learning effects, we relied on randomization. The software presented the four articles in a random sequence, and additionally randomized the order of the two semi-automated and two manual assessments. These randomization strategies are illustrated in Fig. 3.

**Fig. 4:**
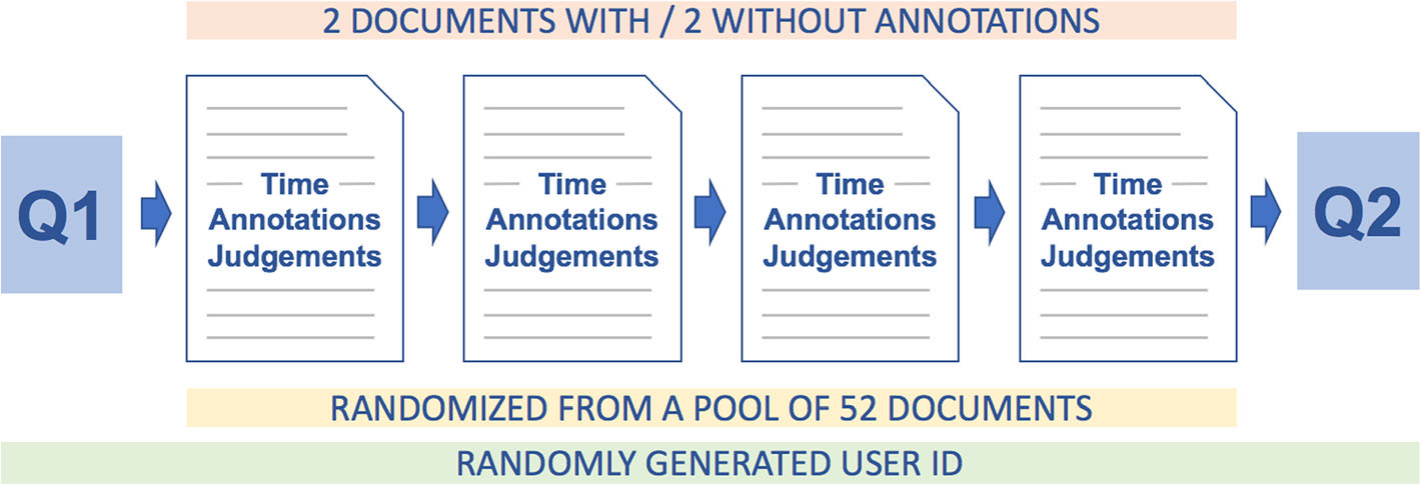
The user study pipeline overview

Participants additionally completed two brief questionnaires: one at the beginning of the study (Q1) which consisted of three questions concerning their level of experience with systematic reviews, and one at the end (Q2) which comprised a usability assessment. The latter is described in more detail below. Figure 4 shows a flowchart of the study and its structure.

### Outcomes

The primary outcome of interest was the time taken to complete bias assessments. We additionally assessed the quality of machine learning suggestions, and asked participants to evaluate the usability of the system.

To assess quality, we compared the initial machine learning suggestions with the final decisions made by the users. We calculated the proportion of judgements (i.e. judgements that articles were at ‘low’ or ‘high/unclear’ risk of bias) that were overturned by the user, and the precision and recall of the machine learning rationales with respect to the user’s final submitted edits.

To evaluate usability, we used the standard System Usability Scale (SUS), and we collected qualitative feedback regarding user experience and subjective perception of system usability. The SUS has been shown to be applicable across many domains and technologies [17, 18]. Drawing upon this work one can use the SUS to place systems “on a scale somewhere between the extremes of dire and wonderful usability” [19].

The SUS includes 10 standard general questions which afford comparisons to the many other systems that have been evaluated with this scale. We added six specific questions focused on the task of bias assessment which we analyze independently.

To avoid response bias (i.e., participants responding uniformly with all ‘5’s or all ‘1’s), the SUS includes some positive questions (“agree” response reflects positive usability assessment) and negative questions (“disagree” response reflects positive usability assessment) (see Table 1).

**Table 1:** Usability questionnaire completed by study participants; each was scored using a Likert scale from 1 (*strongly disagree*) to 5 (*strongly agree*)

Usability Questions	
*System Usability Scale (SUS) Questions*	
I think I would like to use this system frequently.	
I found the system unnecessarily complex.	
I thought the system was easy to use.	
I think that I would need the support of a technical person to be able to use this system.	
I found the various functions in this system were well integrated.	
I thought there was too much inconsistency in this system.	
I would imagine that most people would learn to use this system very quickly.	
I found the system very cumbersome to use.	
I felt very confident using the system.	
I needed to learn a lot of things before I could get going with this system.	
*Additional questions relating to bias assessment*	
I found the text suggested by the computer helpful in completing the task.	
I found it difficult to navigate to the sections of the article suggested as relevant by the model.	
I feel that having the computer suggest text to reviewers would improve the quality of the final output (i.e., the information extracted for the systematic review).	
I felt the text suggested by the computer was often irrelevant.	
I was confused by the text that the computer suggested.	
I would like to continue using this system to aid systematic review production.	

In addition to the structured SUS questions, we asked participants to provide qualitative feedback regarding their user experience via a free-text box presented at the end of the questionnaire.

### Statistical methods

Times taken to conduct assessments were measured in seconds, and the significance of raw differences in times calculated using the Wilcoxon signed rank test.

To account for inter-participant differences in speed (as each participant functioned as their own control), we used a mixed linear model, incorporating the condition (semi-automation vs manual) as a fixed effect, and the participant identifier as a random effect. We transformed times to a logarithmic scale both to ensure that they were normally distributed, and also to allow interpretation of model coefficients as (the logarithm of) relative time differences. Mixed models were estimated in *R* using the *lme4*^7^ package [20].

Articles varied in difficulty, and the ordering of article presentation seemed to have an effect on time taken for assessments. We therefore additionally conducted post-hoc sensitivity analyses to evaluate the effect of these variables on time taken, which are presented in the online Appendix.

### Ethics

This study was approved by the King’s College London BDM Research Ethics Panel, and was deemed exempt from requiring ethical approvals by the IRB committees of Northeastern University and the University of Texas at Austin.

## Results

Forty-one participants were recruited, with a wide range of levels of experience (see Table 2). All except five had completed at least one systematic review, and all but nine were familiar with the Cochrane Risk of Bias tool. 66% of participants had completed 5 or more systematic reviews.

**Table 2:** Self-reported participant characteristics

Participant characteristics	Category	Number (%)
Number of systematic reviews conducted	0	5 (12%)
	1–5	9 (22%)
	5–10	12 (29%)
	10+	15 (37%)
Experience using the Cochrane Risk of Bias tool	yes	32 (78%)
	no	9 (22%)

**Fig. 5:**
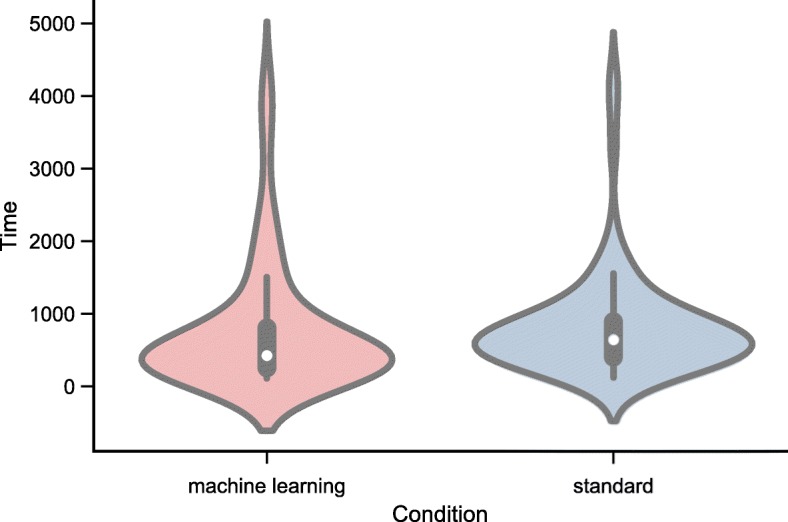
Violin plots of total time (in seconds) taken using the machine learning aided versus standard (fully manual) approach

The mean time taken to assess an article was 755 s (SD 868) for *semi-automated* assessment, and 824 s (SD 717) for *manual* assessment (see Fig. 5 and Table 3 *P* < 0.001). In our mixed model adjusting for participant identifier as a random effect, semi-automation was 25% faster than fully manual bias assessment: relative time for semi-automation vs manual 0.75, 95% CI 0.62 to 0.92.

**Table 3:** Time taken to perform risk of bias assessments using machine learning assisted, and fully manual approaches. Relative time estimated from mixed effects model, adjusting for inter-participant differences as a random effect

	Machine Learning (*n* = 82)	Standard (*n* = 82)	*p* value	Relative Time
Time	mean = 755	mean = 824	*p* < 0.001	0.75 (95% CI 0.62 to
(seconds)	(SD = 868)	(SD = 727)		0.92)

We conducted exploratory analyses to investigate the effects of document ordering and difficulty on the time taken to conduct bias assessment (see online Appendix^8^). These revealed that both affected the time taken for assessments. We thus sequentially explored the effects of these factors via additional mixed effects models, which reduced model residuals, but did not result in important changes to the estimate of relative time reduction achieved via semi-automation. The methods and results of these analyses are presented in detail in the aforementioned online Appendix.

### User edits to the machine learning suggestions

We assess the changes users made in the machine learning assisted articles only (no calculation of change is possible in the fully manual task). Eighty-two articles were assessed with machine-learning assistance (two per participant), and each of these assessments included four bias domains (thus 328 bias domain assessments were conducted in total).

Concerning the bias judgments (i.e. assignment of ‘low’ or ‘high’ risk of bias labels), participants made no change in 301/328 (91%) of bias domain assessments. Rates of change were similar across bias domains (see breakdown in Table 4).

**Table 4:** Quality of machine learning suggestions: proportion of machine learning judgements (i.e. ‘low’ or ‘high/unclear’) overturned by the user

	Overall (*n* = 328)	RSG (*n* = 82)	AC (*n* = 82)	BPP (*n* = 82)	BOA (*n* = 82)
Judgements changed	27 (9%)	7 (9%)	7 (9%)	6 (7%)	7 (9%)

**Fig. 6:**
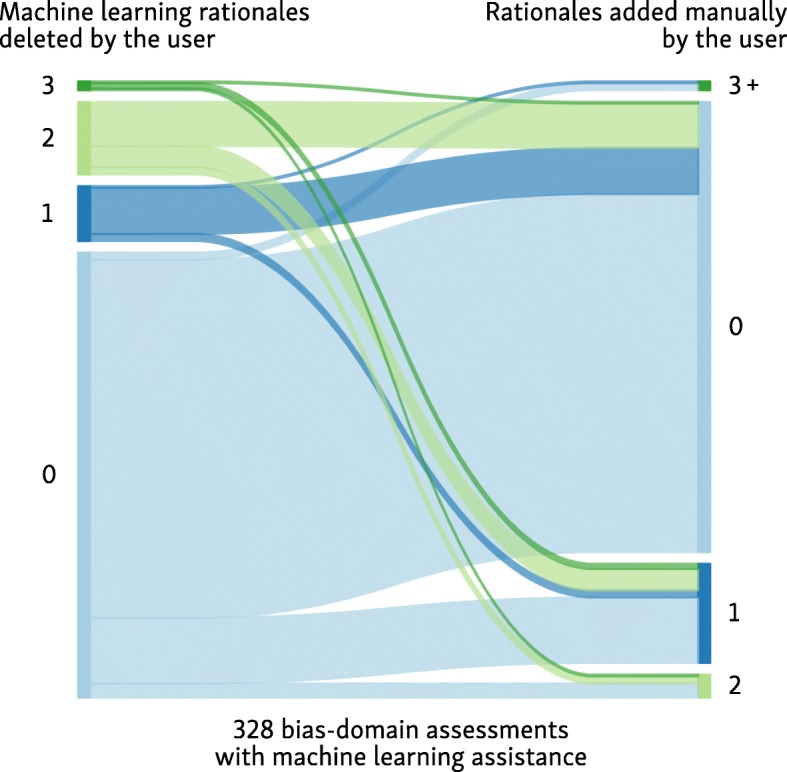
Flow diagram of user interaction with the machine learning suggested rationales (text highlights)

We present a breakdown of the user interactions with the machine learning rationales as a flow diagram in Fig. 6. Participants made no changes to 202 (62%) of suggestions; made partial changes to 113 (35%) of the annotations (i.e., edited some of the suggested text, but left some unchanged), and deleted and replaced all ML-suggested rationales in 13 (4%). On average, ML-suggested rationales had a recall of 0.90, and precision of 0.87 with regard to the finalized user-edited rationales; precision and recall were similar across the bias domains (see Table 5).

**Table 5:** Quality of machine learning suggestions: Precision/recall of the machine learning text highlights with regard to the users’ final edits (calculated for each bias assessment which used machine learning assistance, and averaged)

	Overall (*n* = 328)	RSG (*n* = 82)	AC (*n* = 82)	BPP (*n* = 82)	BOA (*n* = 82)
Recall (SD)	0.90 (0.14)	0.91 (0.18)	0.92 (0.17)	0.90 (0.19)	0.91 (0.21)
Precision (SD)	0.87 (0.21)	0.91 (0.21)	0.88 (0.24)	0.87 (0.25)	0.87 (0.27)

### System usability scale (SUS) scores

**Fig. 7:**
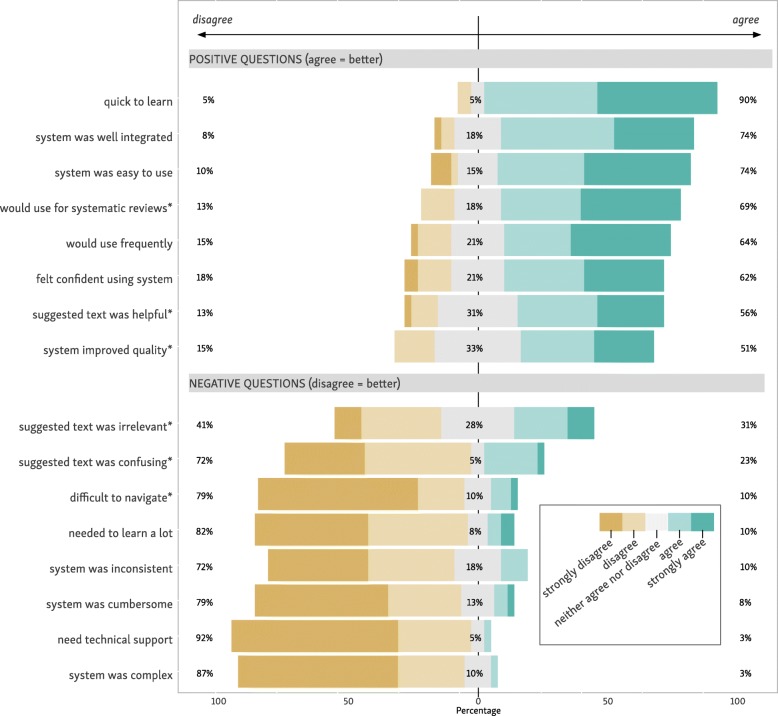
Question-by-question breakdown of the SUS questionnaire responses; *supplementary task-specific questions

For the standard SUS, participants gave a mean score of 77.4 (SD 14.2). Scores of 68 or above are typically regarded as “above average” [21]. A study of the SUS by Bangor and colleagues found that SUS scores were reliably related to verbal descriptions [22]. Under this interpretation, our system usability is somewhere between “good” (mean SUS 71.4), and “excellent” (mean SUS 85.5). A summary of the question-by-question responses is given in Fig. 7.

Regarding our supplementary task-specific questions, 69% of participants “strongly agreed” and “agreed” that they would use our system for systematic reviews. 56% “strongly agreed” or “agreed” that the ML suggested text was helpful. 51% “strongly agreed” or “agreed” that the system improved the quality of assessments. On the negative coded questions, 79% “strongly disagreed” or “disagreed” that the system was difficult to navigate. 72% “strongly disagreed” or “disagreed” that the suggested text was confusing. Finally, 41% stated that they “strongly disagreed” or “disagreed” that the suggested text was irrelevant (see Fig. 7).

### Qualitative feedback

Of the 41 participants, 38 (92.7%) provided qualitative feedback. Illustrative examples of feedback are provided in Table 6. The vast majority of comments concerned practical suggestions for improving the tool interface (e.g., improving the visibility of highlighting color). Other comments highlighted more conceptual concerns about using such technology. For example, a concern that highlighted text might distract reviewers from properly scrutinizing the entire paper. The most frequent pieces of feedback were: a desire to separate the categories of ‘high’ and ‘unclear’ risk of bias (6 participants); addition of a comment box for reviewer’s notes (4 participants); and providing a link to guidance on applying Cochrane Risk of Bias tool (3 participants).

**Table 6:** Illustrative examples of participant qualitative feedback, stratified by SUS score category

Qualitative Feedback	SUS score from user
SUS scores > = 68 (i.e. rated usability as above average)	
We should have 3 risk choices (as it is for Cochrane review): low, unclear and high risk, and not only low versus unclear/high.	97.5
think the suggested text is good, so long as it doesn’t make people lazy.	92.5
The two blinding questions are confusing because the form did not specify which outcome that I am supposed to assess.	90
I had a hard time finding the green highlighted information\dots this colour was hard for me to see	87.5
I think it would be helpful to have a pop-up window that would have the explanation for each of the risk of bias questions.	77.5
SUS scores < 68 (i.e. rated usability as below average)	
Technical problems with highlighting occurred.	67.5
Risk that reviewers would only focus on suggested text, not full text.	65
What was the order of the annotated text when multiple pieces of text were highlighted? Is it sorted by text order or relevance? I hope it’s by relevance!	57.5
The text suggestions usually had at least one relevant sentence (maybe 1 out of 3) for rating so it meant I was unclicking things to be accurate.	57.5
I found myself searching for an “undo” button when I deleted a suggested text spot, then changed my mind.	55

Regarding the accuracy of the underlying ML models, 6 participants commented that many of the annotations were not relevant, and needed to be deleted.^9^ All datasets including a supporting analysis Jupyter Notebook^10^ are available in a GitHub repository.^11^

## Discussion

Prior to this work, RobotReviewer had been evaluated retrospectively, examining predictive accuracy in comparison with a set of gold-standard data. And indeed, this is the way most ML technologies to aid systematic reviewing have been assessed. However, given the rigorous requirements for biomedical evidence synthesis, humans will certainly remain ‘in-the-loop’ for the foreseeable future.

We thus sought to evaluate the use of ML to support human-conducted bias assessment in practice and from the user perspective. To this end we designed and executed a prospective, randomized trial using a prototype tool that integrates our ML models via a semi-automated workflow. We found that semi-automating bias assessment using ML reduced time spent by 25%, compared to a fully manual approach. The system was rated as highly usable, and we collected qualitative feedback regarding user experience, including recommendations to improve the system. If ML is to actually be used by domain experts for applications in EBM specifically and healthcare more generally, we believe more such user studies are needed.

A key question prompting this study is how ML might be used in practice for systematic reviews. Our previous analyses have in effect examined fully automatic machine learning (e.g., predictive accuracy of bias predictions [11] or data extraction technologies [15]), without human involvement. In the case of bias assessment, such analyses have found machine learning accuracy to be good, but still lagging human performance [8]. A smaller scale but prospective evaluation recently conducted independently found that RobotReviewer bias assessments (likewise done fully automatically) had similar agreement as that found between different author groups [23]. While fully automatic and reasonably accurate assessment may have uses (rapid reviews, ranking search results, or clinical question answering), conventional systematic reviews prioritize rigor and accuracy, and are typically conducted on a timescale to match. Semi-automation allows one to benefit from automation without sacrificing reliability, as suggestions are manually checked.

One concern expressed by colleagues is that ML might encourage reviewers to be less thorough, and that some might ‘okay’ automatic suggestions without detailed reading and analysis of the article and underlying trial — a concern mentioned by several participants in their qualitative feedback. However, the quantitative data from this study provided at least some evidence that this did not occur in practice. ML suggestions reduced time taken for assessments (~ 25%); one might expect more dramatic increases in speed if participants were not engaging in substantive analysis. Articles assessed with the aid of ML predictions still took > 12 min to assess, on average. Likewise, participants made edits to the ML-suggested rationales in over one-third of cases, suggesting active engagement and also that there is still room for the quality of ML technology to improve further; they also provide evidence that participants were not automatically accepting ML-suggestions, but rather were meaningfully engaging with the task. While we did not record the reasons for rejecting annotations directly, we obtained very useful information via the aforementioned qualitative feedback. For example, one reviewer stated: “Some of the text was irrelevant to the particular domain - but I’d rather RR picked up some extra text than missed some (over-inclusive is better - I can easily delete what I don’t want!).” Whereas another reviewer didn’t directly disagree with the annotations but felt they were misplaced: “For the last one, allocation concealment, I felt the correct text was highlighted, but put in the wrong box.”

### Strengths and weaknesses

To our knowledge this is the first evaluation of machine learning in evidence synthesis when used in a real-life setting (albeit in a trial setting), with users typical of our target audience.

Our participants were recruited via email and Twitter, completed the study anonymously, and we did not make extensive attempts to verify their status or experience. However, the fact that participants were recruited primarily via professional networks, and that the study link was only sent after an interested party actively sent a plausible email request, together with the evidence of high user engagement during the study (mean of 52 min completing the study) suggests that participants were genuine. Likewise, the qualitative feedback we received was remarkably detailed and beyond our expectations: the overwhelming majority provided feedback on their user experience, and suggestions for improvements. Of note, participants recruited are likely to be atypical in some ways; it seems likely that systematic reviewers who were open to using new technologies and methods would be more likely to ask to participate than those who are skeptical. Our findings might therefore be optimistic in terms of usability and acceptability if applied to systematic reviewers in general.

One limitation of our system is that we did not provide recommendations for the final two standard domains defined in the Cochrane Risk of Bias tool: selective reporting of outcomes and incomplete reporting of data. These domains typically require more sophisticated analysis of the article. While our system is able to produce recommendations for these domains [8], these are based merely on the article text; arguably it is not really possible to make more sophisticated assessments on this basis. A machine learning system to automate these outcomes using a theoretically sound method would require substantial additional technical complexity, which is not feasible in the short term. However, one option is to add these domains back into RobotReviewer to allow users to assess them manually using the same interface (and with no suggestions displayed).

Lastly, practices in bias assessment evolve over time. At the time of writing, a new version of the Cochrane RoB tool is at draft stage; this makes changes to the recommended methods [24]. While the core principles remain unchanged, the new version recommends the use of explicit decision trees to improve the reliability of judgments including use of additional variables not currently assessed by RobotReviewer. We aim to continue to evolve the tool to adapt to such changing methods.

## Conclusions

Machine learning suggestions can be used to support bias assessment for biomedical systematic reviews via a web-interface. We found that using this semi-automated approach resulted in a 25% reduction in the time taken for risk of bias assessment, compared to the fully manual approach. Participants rated the system as very highly usable, appeared to engage meaningfully in checking and editing suggestions made by the system (rather than automatically accepting), and provided qualitative feedback on their user experience.

More generally, we believe ‘human-in-the-loop’ trials, such as this study, will become increasingly important to demonstrate any utility afforded by ML, and will provide a vital step towards getting new technologies used in practice.

## Abbreviations

AC: Allocation Concealment
BDM: Biomedical & Health Sciences, Dentistry, Medicine Ethics Subcommittee
BOA: Blinding of Outcome Assessors
BPP: Blinding of Participants and Personnel
CDSR: Cochrane Database of Systematic Reviews
CI: Confidence Interval
CNN: Convolutional Neural Network
EBM: Evidence-Based Medicine
EPPI: Evidence for Policy & Practice Information and Co-ordinating Centre
IRB: Institutional Review Board
ML: Machine Learning
MRC: Medical Research Council
MT-SVN: Linear Multi-Task Support Vector Machine model variant
NIH: National Institute of Health
NLP: Natural Language Processing
PDF: Portable Document Format
Q1: Question segment 1
Q2: Question segment 2
R: R scientific programming language
RA-CNN: Rational Augmented Convolutional Neural Network
RCTs: Randomized Controlled Trials
RoB: Risk of Bias
RR: RobotReviewer
RSG: Random Sequence Generation
SD: Standard Deviation
SUS: System Usability Scale
SVM: Support Vector Machine
UK: United Kingdom
URL: Uniform Resource Locator (web address)
US NLM: United States National Library of Medicine

## Footnotes

1
https://github.com/ijmarshall/robotreviewer

2
http://community.cochrane.org/help/data-management-tools/cochrane-crowd

3
https://eppi.ioe.ac.uk/cms/

4
http://www.srsm.org

5
www.robotreviewer.net

6
https://www.youtube.com/watch?v=0xwwze83sBs

7 We provide a Jupyter Notebook with the code for all analyses together with the source data collected as a supplement to this article

8
https://github.com/h21k/RRUX

9 This feedback might result from participants expecting the tool to highlight *only* relevant text. In fact, the system is intentionally designed to over-retrieve rationales, always retrieving the top three most likely rationales (to increase the likelihood that at least *one* will be relevant). However, the trade-off of this approach is that clearly irrelevant text may be highlighted (for example, where only one piece of relevant text appears in the article, or due to inaccuracies of prediction on a particular document).

10
https://nbviewer.jupyter.org/github/h21k/RRUX/blob/master/Analysis.ipynb

11
https://github.com/h21k/RRUX
